# Is intracellular pH a clock for mitosis?

**DOI:** 10.1186/1742-4682-10-8

**Published:** 2013-02-12

**Authors:** L John Gagliardi, Daniel H Shain

**Affiliations:** 1 Department of Physics, Rutgers The State University of New Jersey, Camden, NJ, 08102, USA; 2 Department of Biology, Rutgers The State University of New Jersey, Camden, NJ 08102, USA

## Abstract

Experiments have shown that the intracellular pH of many cells rises to a maximum at the onset of mitosis, subsequently decreasing 0.3 to 0.5 pH units by the end of mitosis. This result, and observations that tubulin net charge depends strongly on pH, may be critical for microtubule (MT) dynamics during mitosis. *In vivo* studies demonstrate that MT dynamics is sensitive to pH, with MT growth favored by higher pH values. Therefore it seems likely that the shift from the dominance of microtubule growth during prophase, and to a lesser extent during prometaphase, to a parity between MT polymerization and depolymerization during metaphase chromosome oscillations is a consequence of gradually decreasing intracellular pH during mitosis. Thus the timing and sequencing of prophase, prometaphase, and metaphase chromosome motions may be understood as an increase in the MT disassembly to assembly probability ratio resulting from a continuously declining intracellular pH.

## Introduction

In the cytoplasmic medium (cytosol) within biological cells, it is generally thought that electrostatic fields are subject to strong attenuation by screening with oppositely charged ions (counterion screening), decreasing exponentially to much smaller values over a distance of several *Debye lengths*. The Debye length (distance over which the electric field decreases to approximately 36.8 % of the previous value) within cells is typically given as 1 nm [1], and since eukaryotic cells have much larger dimensions one is tempted to conclude that electrostatics is not a major factor in explaining mitotic chromosome movements. However, the presence of microtubules, as well as other factors discussed below, force this assumption to be reconsidered.

The characteristics of microtubule lengthening (polymerization) and shortening (depolymerization) follow a pattern known as “dynamic instability”: that is, at any given instant some microtubules are growing while others are undergoing rapid breakdown. In general, the rate at which microtubules undergo net assembly – or disassembly – varies with mitotic stage [2]. Changes in microtubule dynamics are integral to changes in chromosome motions during mitotic stages. Poleward and antipoleward chromosome motions occur during prometaphase and metaphase. Antipoleward motions dominate during the *congressional* movement of chromosomes to the cell *equator*, and poleward motion prevails during anaphase A. It is assumed here that poleward chromosome motions are in response to disassembling kinetochore microtubules at kinetochores and poles, and argued elsewhere [3] that antipoleward chromosome motions are best explained by assembling microtubules at chromosome arms.

Experiments have shown that intracellular pH (pH_i_) of many cells rises to a maximum at the onset of mitosis, subsequently falling steadily through cell division. Although it is experimentally difficult to resolve the starting time for the beginning of pH_i_ decrease during the cell cycle, it appears to drop 0.3 to 0.5 pH units from typical peak values of 7.3 to 7.5 measured during prophase when microtubule polymerization is favored [4,5].

Studies have shown that *in vivo* microtubule polymerization is favored by higher pH values [6], in contrast with *in vitro* studies which suggest a pH optimum in the range of 6.3 to 6.9. The disagreement between these values has been considered in relation to the nucleation potential of microtubule organizing centers like centrosomes [6], suggesting that pH_i_ regulates the nucleation potential of microtubule organizing centers [7,8]. Experiments have also shown that ionic concentrations play an important role in microtubule polymerization [9]. Taken together, these observations seem to favor the more complex physiology of *in vivo* analyses to resolve this question.

Cellular electrostatics is strongly influenced by reduced counterion screening due to layered water adhering to charged molecules. Such water layering – with consequent reduction or elimination of Debye screening – at charged proteins has long been theorized [10,11] and has been confirmed experimentally [12]. Additionally, water between sufficiently close (up to 3 nm) charged proteins has a dielectric permittivity that is considerably reduced from the *bulk* value far from charged surfaces [13-15]. The combination of these two effects (or conditions) – water layering and reduced dielectric constant – can influence cellular electrostatics in a number of important ways. This is especially true in relation to mitosis [15]. For example, these conditions further increase the tendency for an electrostatic assist to aster and spindle self-assembly (see below).

A number of investigations have focused on the electrostatic properties of microtubule tubulin subunits [16-19]. Large scale calculations of tubulin have been conducted using molecular dynamics programs along with protein parameter sets [20]. The dipole moment of tubulin has been calculated to be as large as 1,800 Debye units (D) [17,21]. In experiments conducted near physiological conditions, the dipole moment was 36 D [22], corresponding to a dipole charge of approximately 0.1 electron per dimer. Experiments have shown that tubulin net charge depends strongly on pH, varying quite linearly from −12 to −28 (electron charges) between pH 5.5 and 8.0 [21,23]. This could be important for microtubule dynamics during mitosis because, as noted above, many cell types exhibit a decrease of 0.3 to 0.5 pH units during mitosis.

Tubulin has a large overall negative charge of 20 at pH 7 and as much as 40% of the charge resides on the C-termini, which extend outward from the microtubule axis as a function of pH_i_ (e.g., 4–5 nm at pH_i_ 7 [20]). It seems likely therefore that an increased tubulin charge and the resulting greater extension of C-termini may be integral to an increased probability for microtubule assembly during prophase when pH_i_ is highest [5].

### Intracellular pH as a clock for mitosis

In addition to addressing force generation for post-attachment chromosome motions [3,15,24], a continuum electrostatics approach to mitotic motions can also account for the timing and sequencing of the detailed changes in these motions. These can be attributed to changes in microtubule dynamics based on a progressively increasing microtubule disassembly to assembly ratio for kinetochore microtubules that is caused by a steadily decreasing pH_i_ during mitosis.

It therefore seems reasonable to expect that prophase high pH_i_ conditions and the electrostatic nature of tubulin dimer subunits greatly assists in their self-assembly into the microtubules of the asters and spindle. As pHi increases beyond interphase the presence of nucleating centers, along with the favoring of microtubule polymerization in a higher pHi environment, suggests that the pool of tubulin from interphase microtubule disassembly will polymerize around prophase centrosomes. As discussed in the previous section, this self-assembly would be aided by reduced counterion screening due to layered water and the reduced dielectric constant between charged protein surfaces. An electrostatic component to the biochemistry of the microtubules in assembling asters is consistent with experimental observations of pH effects on microtubule assembly [6], as well as the sensitivity of microtubule stability to calcium ion concentrations [25,26].

The two effects (or conditions) discussed above are expected to increase the efficiency of microtubule self -assembly in asters and spindles by (1) allowing electrostatic interactions over greater distances than Debye (counterion) screening dictates, and (2) further increasing the strength of these interactions by an order of magnitude due to a corresponding order of magnitude reduction in the cytosolic dielectric constant between charged protein surfaces separated by critical distances.

Thus it seems reasonable to assume that, over distances consistent with the reduced dielectric constant and modified counterion screening, the electrostatic nature of tubulin dimers allows tubulin dimer microtubule subunits to align end-to-end and laterally, facilitating the formation of asters and mitotic spindles [24,27].

**Figure 1:**
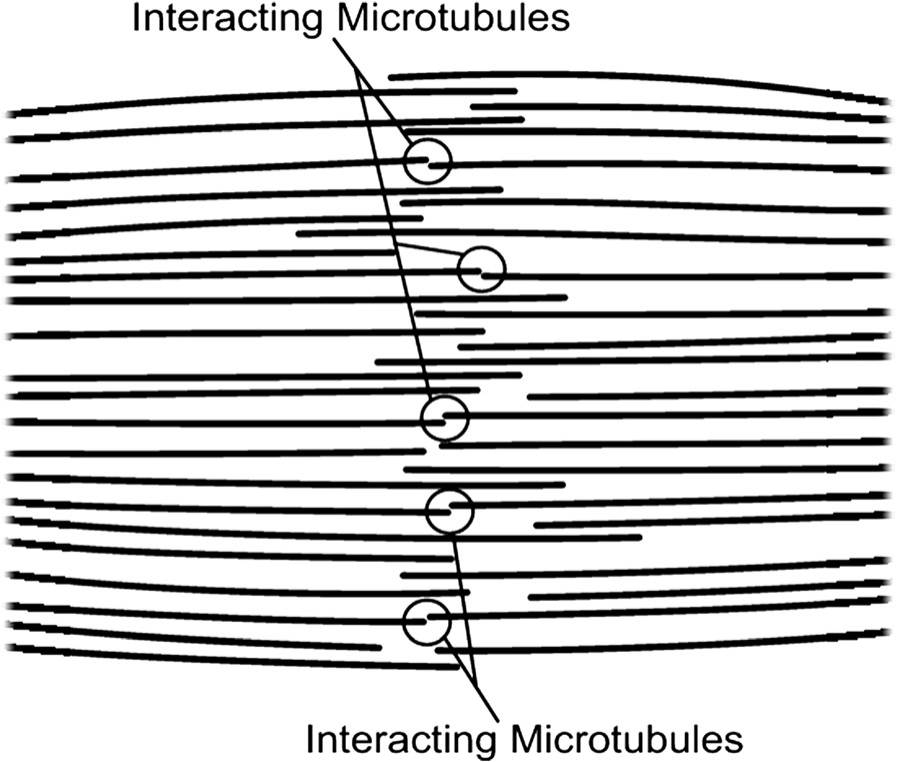
**A subset of interacting microtubules in a small portion of a forming mitotic spindle.** Protofilament curling for disassembling microtubules is not shown on this scale.

Similarly, a mutually repulsive electrostatic force between subsets of like-charged free plus ends of interacting microtubules from opposite half-spindles in the growing mitotic spindle is expected to increase in magnitude and range. Thus mutual electrostatic repulsion of negatively charged microtubule plus ends distal to centrosomes in assembling asters/half-spindles could provide the driving force for their poleward migration in the forming spindle [15,27]. A subset of interacting microtubules in a small portion of a forming spindle is depicted in Figure 1.

Interacting microtubules can result from either growing or shrinking microtubules but polymerization probabilities will dominate during prophase. An increased probability for microtubule depolymerization, as compared to the prophase predominance of microtubule assembly, is consistent with experimental observations of alternating poleward and antipoleward motions – with antipoleward motions more probable – of *monovalently* attached chromosomes during prometaphase. As discussed elsewhere [3,24], after a *bivalent* attachment to both poles, electrostatic poleward forces toward both poles acting in conjunction with inverse square antipoleward forces exerted between negatively charged microtubule free plus ends and negatively charged chromosome arms could account for chromosome congression. Metaphase chromosome mid-cell oscillations are indirect experimental evidence for a microtubule disassembly to assembly (disassembly/assembly) probability ratio approaching unity as pH_i_ continues to decline.

At late metaphase, before anaphase-A, experiments reveal that the poleward motions of sister kinetochores stretch the intervening centromeric chromatin, producing high kinetochore tensions [28]. These high tensions are likely attributed to a continuing microtubule disassembly/assembly probability ratio increase caused by a continuously lowering pH_i_. The resulting attendant increase in poleward disassembly force on sister chromatids would lead to increased tension.

Regarding post-attachment chromosome motions through metaphase, it seems reasonable to ascribe an increasing dissassembly/assembly probability ratio – with attendant changes in microtubule dynamics and associated mitotic chromosome motions through metaphase – to an experimentally-observed steadily decreasing pH_i_. We may then envision a decrease in pH_i_ from a peak at prophase favoring microtubule assembly, declining through prometaphase and continuing to decline through metaphase when parity between microtubule assembly and disassembly leads to mid-cell chromatid pair oscillation, culminating in increased microtubule disassembly-associated kinetochore tension late in metaphase, as the cell’s master clock controlling microtubule dynamics, and consequently the events of mitosis. One might also be tempted to attribute the more complete dominance of microtubule disassembly – with an accompanying predominance of poleward disassembly forces at kinetochores and poles – during anaphase-A to a further continuation of decreasing intracellular pH. However, as discussed elsewhere [3,15], any additional lowering of pH_i_ after metaphase may work in conjunction with increased [Ca^2+^[29,30] as major determinants of anaphase-A and anaphase-B motions.

## Conclusions

High pH_i_ during prophase favors spindle assembly. This includes greater electrostatic attractive forces between tubulin dimers as well as increased repulsive electrostatic interactions between growing microtubule plus ends driving poleward movement of forming half-spindles. Due to reduced counterion screening and the low dielectric constant of layered water adhering to charged tubulin dimers, the necessary attraction and alignment of tubulin dimers during spindle self-assembly would be enhanced by the considerably increased range and strength of electrostatic attractions between oppositely charged regions.

Changes in microtubule dynamics are integral to changes in chromosome motions during mitosis, and can be attributed to an associated change in intracellular pH (pH_i_). In particular, a decrease in pH_i_ through mitosis may act as a master clock controlling microtubule disassembly/assembly probability ratios by altering the electrostatic interactions of tubulin dimers. This, in turn, would determine the timing and dynamics of post-attachment mitotic chromosome motions through metaphase.

Thus it seems reasonable to assume that the shift from the dominance of microtubule growth during prophase, to a lesser extent during prometaphase, and to approximate parity between microtubule polymerization and depolymerization during metaphase chromosome oscillations, can be attributed to the gradual downward pH_i_ shift during mitosis that is observed in many eukaryotic cells.

## Competing interests

The authors declare that they have no competing interests.

## Authors’ contributions

LJG conceptualized the theoretical aspects of this article and DHS provided intellectual contributions. Both authors read and approved the final manuscript.
